# Quantum Behavior of Spin-Orbit Inelastic Scattering of C-Atoms by D_2_ at Low Energy

**DOI:** 10.3389/fchem.2019.00164

**Published:** 2019-03-28

**Authors:** Astrid Bergeat, Sébastien B. Morales, Christian Naulin, Jacek Kłos, François Lique

**Affiliations:** ^1^Univ. Bordeaux, CNRS, ISM, UMR 5255, Talence, France; ^2^Department of Chemistry and Biochemistry, University of Maryland, College Park, MD, United States; ^3^LOMC - UMR 6294, CNRS-Université du Havre, Le Havre, France

**Keywords:** chemical physics, dynamics of molecular collisions, inelastic collisions, astrochemistry, crossed-molecular beam, theoretical calculations, spin-orbit excitation

## Abstract

Fine-structure populations and collision–induced energy transfer in atoms are of interest for many fields, from combustion to astrophysics. In particular, neutral carbon atoms are known to play a role in interstellar media, either as probes of physical conditions (ground state ^3^P_*j*_ spin-orbit populations), or as cooling agent (collisional excitation followed by radiative decay). This work aims at investigating the spin-orbit excitation of atomic carbon in its ground electronic state due to collisions with molecular deuterium, an isotopic variant of H_2_, the most abundant molecule in the interstellar medium. Spin-orbit excitations of C(^3^P_*j*_) by H_2_ or D_2_ are governed by non-adiabatic and spin-orbit couplings, which make the theoretical treatment challenging, since the Born-Oppenheimer approximation no longer holds. Inelastic collisional cross-sections were determined for the C(^3^P_0_) + D_2_ → C(^3^P_*j*_) + D_2_ (with *j* = 1 and 2) excitation process. Experimental data were acquired in a crossed beam experiment at low collision energies, down to the excitation thresholds (at 16.42 and 43.41 cm^−1^, respectively). C-atoms were produced mainly in their ground spin-orbit state, ^3^P_0_, by dissociation of CO in a dielectric discharge through an Even-Lavie pulsed valve. The C-atom beam was crossed with a D_2_ beam from a second valve. The state-to-state cross-sections were derived from the C(^3^P_*j*_) (*j* = 1 or 2) signal measured as a function of the beam crossing angle, i.e., as a function of the collision energy. The results show different quantum behaviors for excitation to C(^3^P_1_) or C(^3^P_2_) when C(^3^P_0_) collides with *ortho*-D_2_ or *normal*-D_2_. These experimental results are analyzed and discussed in the light of highly accurate quantum calculations. A good agreement between experimental and theoretical results is found. The present data are compared with those obtained for the C-He and C-H_2_ collisional systems to get new insights into the dynamics of collision induced spin-orbit excitation/relaxation of atomic carbon.

## Introduction

Processes controlling internal state population distributions of atomic or molecular species are of interest in many fields. This is especially the case for astrophysics. In particular, information concerning the physical conditions prevailing in interstellar media can only be retrieved from spectra yielding the internal states relative populations of the observed species, which result from the equilibrium between collisional and radiative processes. For that purpose, neutral carbon atoms are of particular interest, since they are known to play a role in interstellar media, either as probes of physical conditions (ground state ^3^P_*j*_ spin-orbit populations), or as a cooling agent (collisional excitation followed by a radiative decay). Indeed, C atoms are very abundant in many interstellar regions ranging from dense molecular clouds to planetary nebulae (Zmuidzinas et al., 1988; Bensby and Feltzing, 2006; Herbst and Yates, 2013). Hence, modeling the fine-structure population of C atoms is of major interest for astrochemical models. Such models rely on both collisional and radiative data. Collisional cross-sections and rate coefficients implying the C atoms and the dominant collisional partners in the interstellar medium (H_2_, He, H) have then to be determined at low temperatures. However, when molecular collisions (reactive or inelastic) occur at low energy/temperature, in the *near cold* regime (1–50 K), resonances are predicted by theory for many systems. Such resonances have been clearly observed in the collision energy dependent integral cross-sections (ICS) for spin-orbit excitation of C(^3^P_0_) by collisions with He (Bergeat et al., 2018) and H_2_ (Kłos et al., 2018). These processes are governed by non-adiabatic and spin-orbit couplings, which makes the theoretical treatment challenging since the Born-Oppenheimer (Born and Oppenheimer, 1927) approximation no longer holds. The challenge was successfully overcome for the C(^3^P)+He two-atomic system (Bergeat et al., 2018): the excellent agreement between experimental results and theory allowed a detailed description of resonance features by theory to be obtained. The situation was quite more complex for the C(^3^P)+H_2_ three-atomic system (Kłos et al., 2018), where multiple diabatic potential energy surfaces (PESs) along with spin-orbit and non-adiabatic couplings are necessary to describe its dynamics. A good agreement was also found, not sufficient however to allow for a clear assignment of resonances involving too great of a number of partial waves. Moreover, at higher collision energies, an intersystem crossing may occur with the PESs emerging from the C(^1^D)+H_2_ insertion reaction. In the energy regions close to the intersystem crossing, one can expect, that the use of only triplet PESs will be too approximate to describe the collisional excitation of the C atom, which was not investigated in the study of C+H_2_ collisions (Kłos et al., 2018). Moreover, most recently, Shen et al. (2017) elucidated importance of the stationary points, in particular the so-called van der Waals saddles in the entrance channel region of the C(^1^D)-H_2_ reaction. Therefore, it is also of interest to confirm the characterization of the entrance channel in the C(^3^P)-H_2_ system. Our experiments will allow to test the C(^3^P)-H_2_ PESs in the lower region of collision energies.

In order to get a clearer insight into the latter system, new experiments and calculations have been performed on the isotopolog system so that the C(^3^P_0_) + D_2_ → C(^3^P_*j*_) + D_2_ (with *j* = 1 and 2) collisional process have been studied. Measurements of state-to-state cross-sections as a function of the collision energy for C(^3^P)-D_2_ scattering have been achieved, using the same apparatus as the one described in our previous studies on C(^3^P)-He (H_2_) inelastic collisions. Quantum dynamical calculations were performed with the same PESs as for C(^3^P)-H_2_ system and differing by the reactant reduced mass and rotational constant of D_2_: it thus provides a further test of these potential energy surfaces that are mandatory to accurately describe and understand the dynamics of the title system.

## Methods

### Experiment

Integral cross-sections were measured using a crossed beam apparatus, with variable crossing angle. The C and D_2_ beams with low velocities and high velocity resolution (see Table 1) were obtained using two cryogenically cooled Even-Lavie pulsed valves (Pentlehner et al., 2009) and collided at a beam intersection angle which could be continuously varied from 90° to 12.5° (Chefdeville et al., 2012). C-atoms were produced by dissociation of CO diluted in neon in a dielectric barrier discharge incorporated in the faceplate of the valve (Even, 2015). *ortho*-D_2_ (hereafter *o-*D_2_) was produced after spin-conversion in a cryogenic cell by liquefaction of *normal*-D_2_ (hereafter *n-*D_2_) on NiSO_4_ catalyst at low temperature (<20 K), every 45 min. The *n*-D_2_ and *o*-D_2_ beam velocities were changed by adjusting the temperature of the valve cold head (see Table 1 for the different experimental conditions).

**Table 1:** Characteristics of the molecular beams used in the determination of the integral cross-sections.

**Beam**	**Gas mixture**	***T*^a^** **(K)**	***v*^b^** **(ms^**−1**^)**	**δ*v*_HWE_^b^** **(ms^−1^)**	$\tau_{0}^{\text{c}}$ **(μs)**	**δα^d^** **(°)**
#1 C(^3^P)	CO:0.3%/Ne	300	815 ± 11	13.5	8.6	1.1
#2 *o*-D_2_ or *n*-D_2_	Neat	100 (117)	1180 ± 19	53	9.9	1.69
#3 *o*-D_2_	Neat	45 (68)	855 ± 20	36.9	11	1.69
#3 *n*-D_2_	Neat	45 (68)	845 ± 12	36.9	11	1.69
#4 *o*-D_2_	Neat	10 (47)	694 ± 10	38	12.9	1.65

a *Temperature set point of the cold head. The effective temperature of the pulsed valve is limited by losses due to thermal radiation. The effective nozzle temperatures deduced from the He beam velocity are given in parentheses*.

b *Beam velocity peak values and spreads were deduced from temporal profiles recorded at the crossing point by REMPI detection, and at d = 393.3 mm downstream, with a fast-ionization gauge (FIG) inserted perpendicular to the molecular beam*.

c *Pulse duration at the crossing point (hwe) deduced from the REMPI signals*.

d *Angular divergence (HWE)*.

The collision energy, *E*_*T*_, in our crossed beam machine was varied by changing the angle between the two supersonic beams from χ = 90° to 20 or 13°: $E_{T}=\frac{1}{2}\mu \left\{v_{C}^{2}+v_{D_{2}}^{2}-2v_{C}v_{D_{2}}cos\chi \right\}$.

**Figure 1:**
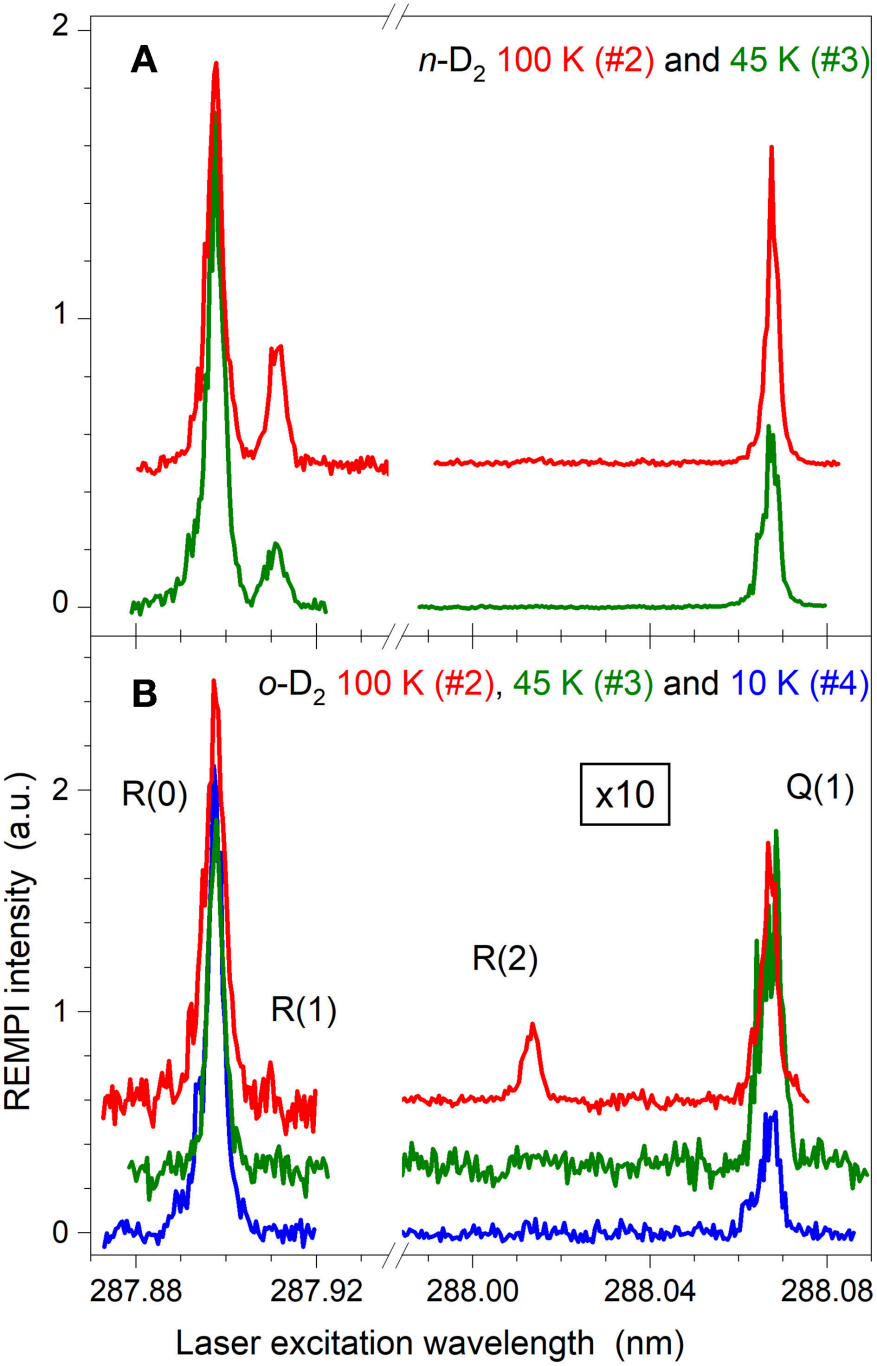
(3+1) REMPI spectra of *n*-D_2_ **(A)** and *o*-D_2_ **(B)** at crossing point for different temperature set points of the cold head. Note that for *o*-D_2_ **(B)** the scale of the right part is magnified by a factor of 10. Temperatures (100, 45, and 10 K) are the set point of the cold head and correspond to the experimental conditions #2, #3, and #4 given in Table 1.

The carbon atoms were probed in their various spin-orbit states via (2+1) resonance-enhanced multiphoton ionization (REMPI) technique, using 2-photon 2p^2^ ^3^P_*j*_ → 2p3p ^3^P_*j*_ transitions (excitation laser tuned at 280.3 nm) at 140.149 nm, 140.157 nm and 140.170 nm for the C(^3^P_0_), C(^3^P_1_) and C(^3^P_2_), respectively (Geppert et al., 2003). A third photon ionized the carbon atoms and C^+^ is detected by a time-of-flight (TOF) mass spectrometer. Populations of *ca* 90–96% for C(^3^P_0_), 4–10% for C(^3^P_1_), and < 1% for C(^3^P_2_) were deduced from the intensities of these transitions, consistently with previous studies (Bergeat et al., 2018; Kłos et al., 2018). The laser beam was perpendicular to the crossing beams plane, hence avoiding any Doppler shift while scanning the crossing angle, χ, for the ICS measurements. Spectra obtained by 3+1 REMPI on the transitions $C^{1}\Pi_{u}\left(v^{\prime }=3,j^{\prime }\right)\leftarrow X^{1}\Sigma_{g}^{+}\left(v^{\prime \prime }=0,j^{\prime \prime }\right)$ were employed to detect individual D_2_ rotational levels (see Figure 1). Only the lowest rotational level of each nuclear spin modification, i.e., *j*_*D*_2__ = 0 and 1 for *o*-D_2_ and *p*-D_2_, respectively, was found to be populated in the supersonic beams. The only exception was for *o*-D_2_, 100 K: a small signal can be seen for the R(2) transition. The *ortho* converted-D_2_ was found to contain <6% *para*-D_2_. These values were found to change inappreciably over the D_2_ velocities (or the temperature of the valve) and over 1.5 h.

**Figure 2:**
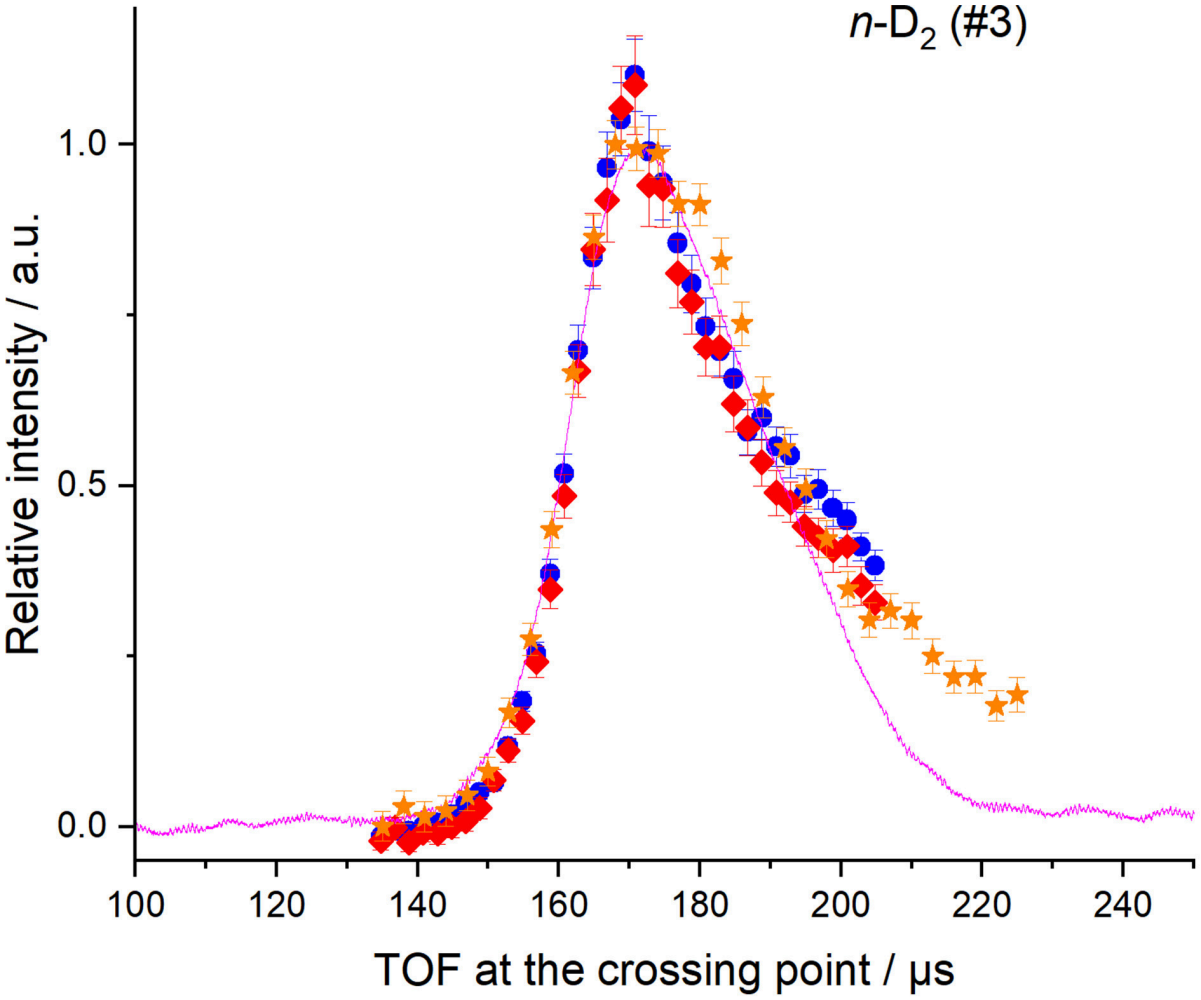
REMPI or FIG profiles at crossing point, of *n*-D_2_ (for a cold head at 45 K). D_2_ molecules in the rotational levels *j* = 0 and 1, were detected using the R(0) and R(1) transitions (blue circles and red diamonds); C(^3^P_2_) atoms, produced by collisions with the D_2_ beam at 70° of the C beam were also detected (orange stars). Vertical error bars correspond to statistical uncertainties of 50 laser shots at 95% of the confidence interval. FIG signal of the D_2_ beam was also recorded at the crossing point using a small skimmer of 1.26 mm aperture in front of the FIG (magenta solid line) and is the average of 128 measurements done by the scope.

**Figure 3:**
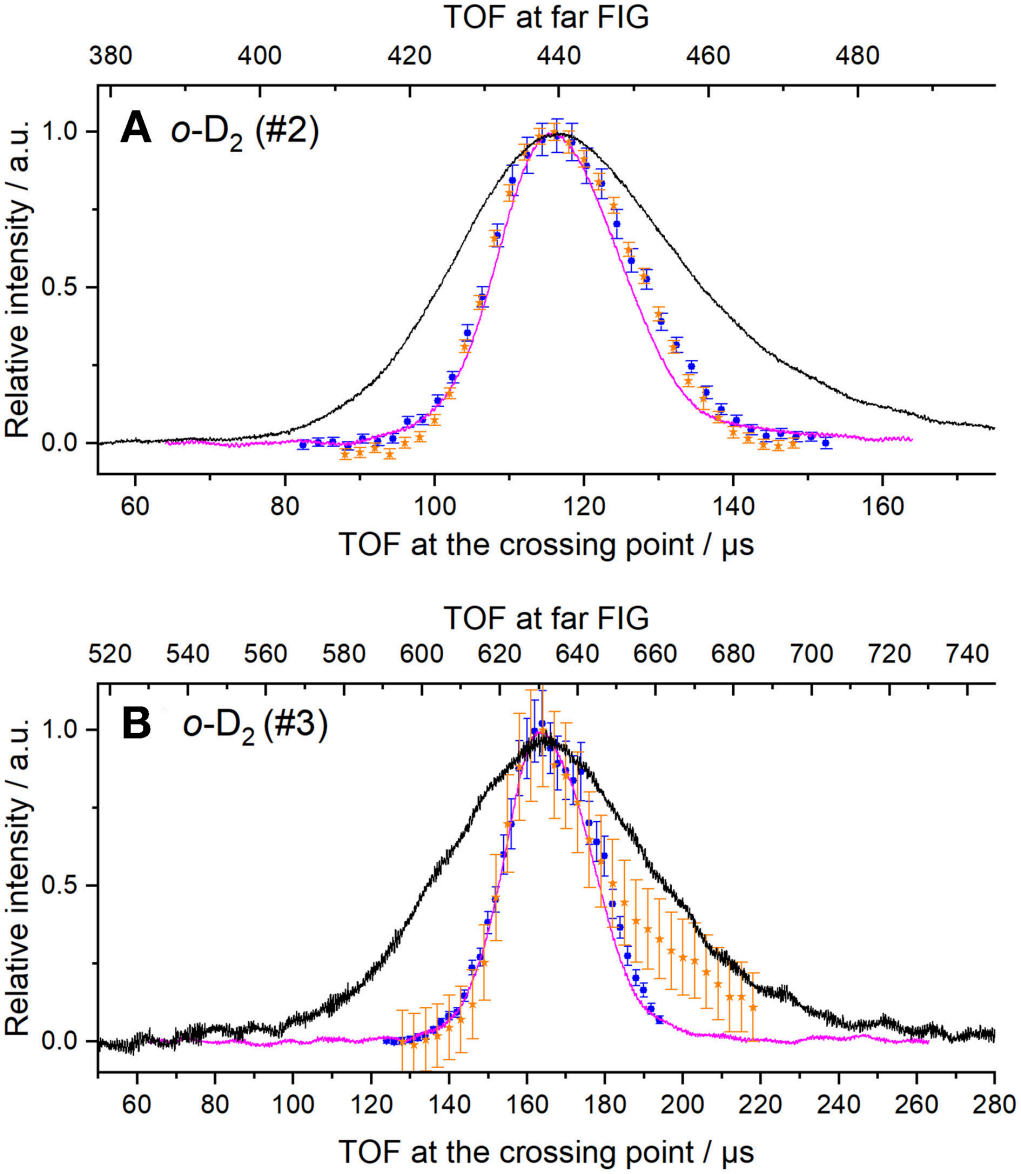
REMPI or FIG profiles of *n*-D_2_ at crossing point and at *d* = 393.3 mm downstream under two experimental conditions [**(A)** #2 and **(B)** #3]. Lower TOF scale: D_2_ molecules in the rotational levels *j* = 0, were detected using the R(0) transition (blue circles); C(^3^P_2_) atoms, produced by collisions with the D_2_ beam at 70° of the C beam were also detected (orange stars). Vertical error bars correspond to statistical uncertainties of 50 laser shots at 95% of the confidence interval. FIG signal of the D_2_ beam was also recorded at the crossing point using a small skimmer of 1.26 mm aperture in front of the FIG (magenta solid line) and is the average of 128 measurements done by the scope. Upper TOF scale: far FIG signal of the D_2_ beam was also recorded at 393.3 mm downstream using a screen with a hole of 6mm-diameter in front of the far FIG (black solid line).

**Figure 4:**
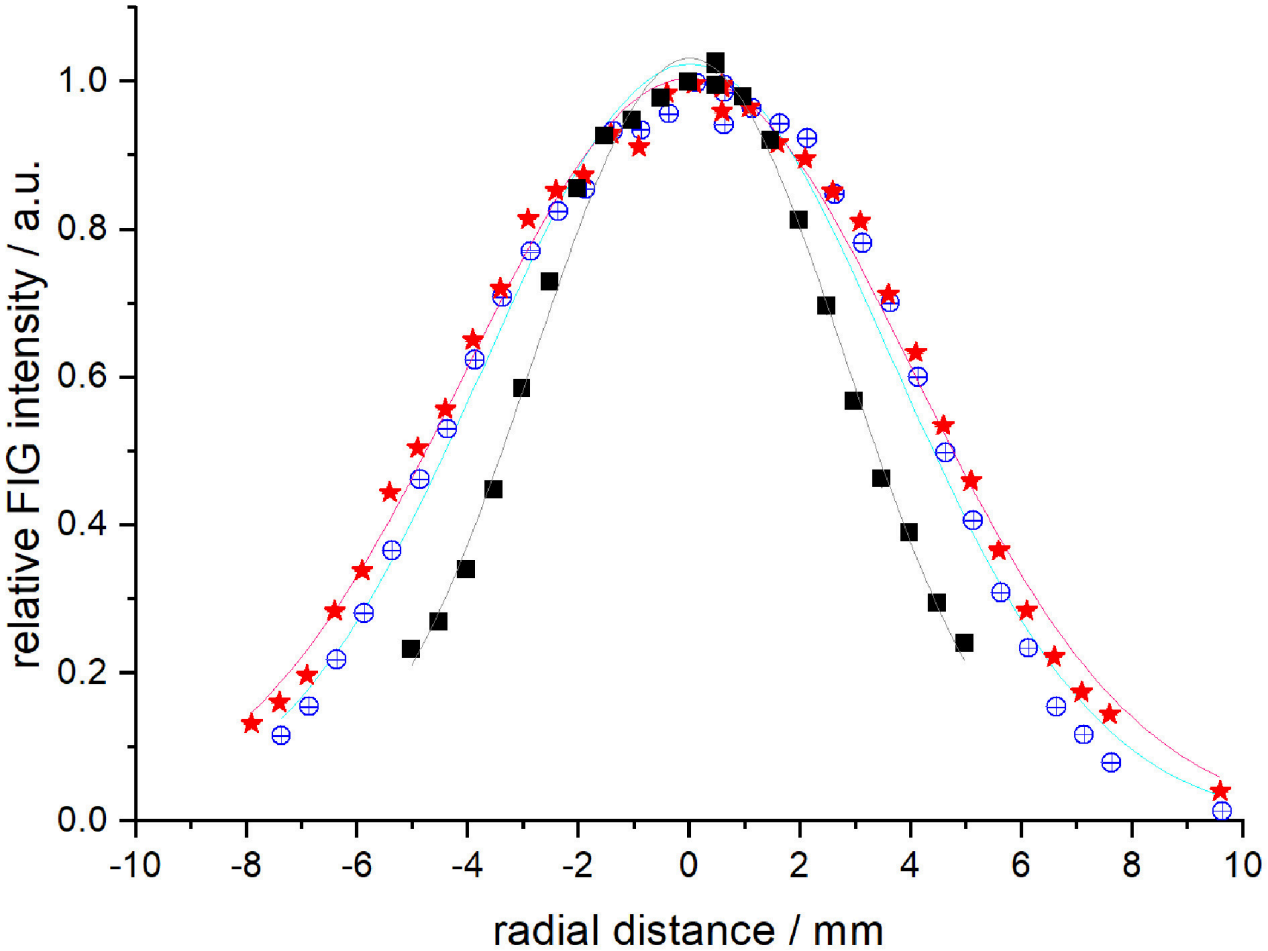
Evolution of the maximum intensities at crossing point measured with the FIG. Black squares: *n*-D_2_ beam under experimental condition #2 with a distance from the valve to the crossing point of 138.7 mm. Blue open circles: Ne beam with a distance from the valve to the crossing point of 350.94 mm. Red stars, metastable Ne^*^ probed with a null current of the FIG.

The beam velocities and spreads (Table 1) were deduced from temporal profiles recorded at the crossing point by REMPI detection or with a fast ionization gauge (FIG) inserted perpendicular to the molecular beam, and at *d* = 393.3 mm downstream, with a second FIG. D_2_ can be detected with the FIG, and by REMPI at the crossing point but not during the ICS measurements: it was then detected indirectly via the excited carbon C(^3^P_1 or 2_) produced by collisions of C atoms with D_2_. In the latter case, the trigger delay of the D_2_ beam for the ICS measurements was adjusted to obtain a maximum REMPI signal of carbon atoms in the (^3^P_1_) or (^3^P_2_) states. Typical REMPI profiles obtained for D_2_ produced in the experimental condition #3 where the cold head temperature was set at 45 K (Table 1) are given in Figure 2. To confirm the beam profiles obtained indirectly during the ICS measurements, two other experiments were also performed independently with the REMPI technique directly on D_2_ molecules (see Figure 1) or with a FIG inserted perpendicular to the molecular beam. Typical results of time-of-flight (TOF) profiles are given Figure 2 for *n*-D_2_ beam in the experimental conditions #3. All the profiles of D_2_ (*j*_*D*_2__ = 0 and 1) may be superimposed: only the intensities differ due to the population distribution, which means that all the rotational states are generated during the expansion and uniformly spread into the beam of *n*-D_2_. Once the profile at the crossing point achieved, the D_2_ beam arrival time, measured with the FIG at *d* = 393.3 mm downstream, yielded the D_2_ velocity. All profiles were fitted to Gaussian functions, with peak positions *t*_0_ and *t*_1_ and half-width at 1/e (hwe), yielding the peak velocities *v* = *d* / (*t*_1_ – *t*_0_), and the velocity spreads δ*v*_hwe_ from the pulse broadening (Naulin and Bergeat, 2018). The response time of the FIG was previously determined to be ~3 μs [see Supplementary Materials of Chefdeville et al. (2013)], and *t*_1_ values are thus accordingly corrected. With this method, the velocities of the D_2_ beam were checked, before, during and after the ICS measurements of each day to ensure the absence of any drift. Typical results for *o*-D_2_ generated under the conditions #2 and #4 are given in Figure 3. To determine the angular spread of the beam, the FIG at the crossing point, mounted on a translation stage, was moved perpendicularly to the beam axis to enable measurements at various positions. A depth gauge allows the exact re-positioning of the FIG. It should be noted that the TOF profiles don't change with the position of the FIG, which means that the transverse velocity is negligible compared to the beam velocity. The area of the TOF profiles or the maximum intensity varies with the position of the FIG: the angular spreads given in Table 1 were determined by fitting the curves with Gaussian functions (see Figure 4).

**Figure 5:**
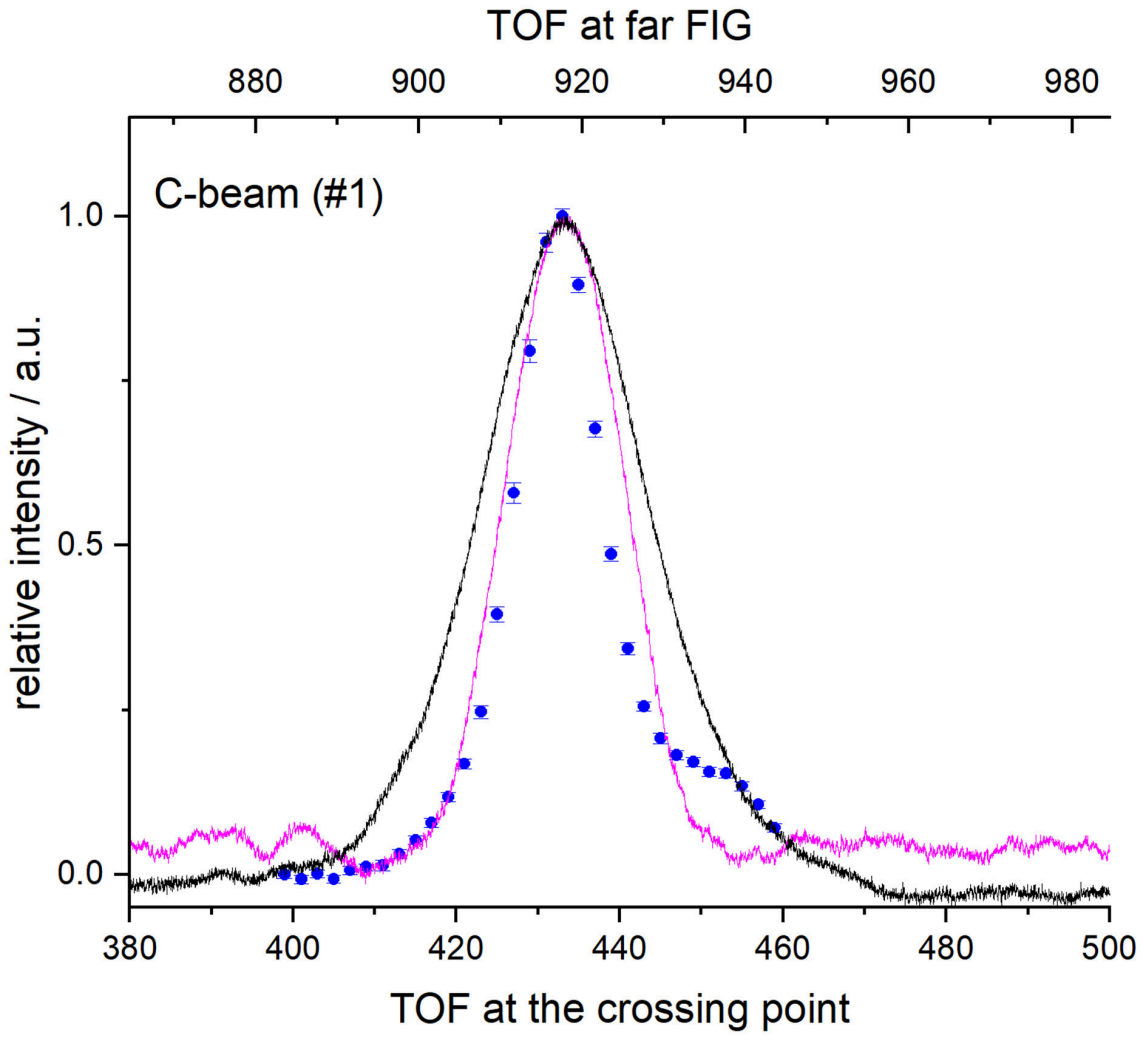
REMPI or FIG profiles of C and metastable Ne^*^ at crossing point and at *d* = 393.3 mm downstream under two experimental conditions #1. Lower TOF scale: C(^3^P_0_) atoms were detected by (2+1) REMPI technique at 140.149 nm. Vertical error bars correspond to statistical uncertainties of 50 laser shots at 95% of the confidence interval. FIG signal of Ne^*^ produced in the C-beam was also recorded at the crossing point using a small skimmer of 1.26 mm aperture in front of the FIG (magenta solid line) and a null current in the FIG. Each profile is the average of 128 measurements done by the scope. Upper TOF scale: Ne^*^ far-FIG signal recorded at 393.3 mm downstream using a screen with a hole of 6mm-diameter in front of the far FIG (black solid line).

Carbon atoms can only be detected by REMPI at the crossing point, but not with the FIG, contrarily to metastable Ne^*^ atoms also generated within the discharge. They give a signal due to surface ionization with the FIG operated with a null filament current. Since C(^3^P) and Ne^*^ profiles obtained by REMPI at the crossing point are very similar, it was assumed that their velocities were likely to be identical. Again, beam profiles could also be measured at the crossing point when replacing the mass spectrometer by a FIG. The two profiles obtained for metastable Ne^*^ are identical (see Figure 5). As for D_2_, Ne^*^ velocity values and spreads could be determined from profiles at the crossing point and at *d* = 393.3 mm downstream.

ICSs were obtained from the averaged REMPI signal intensities *I*_REMPI_ and the relative velocity *v*_r_ of the C and D_2_ beams as *I*_REMPI_/(*v*_*r*_ 〈Δ*t*〉), where 〈Δ*t*〉 is the mean interaction time between the two beam pulses which takes full account of the density-to-flux transformation under our working conditions (Naulin and Costes, 2014), and assuming a forward angular distribution of scattered C-atoms as for the C-H_2_ system (Kłos et al., 2018). To monitor the efficiency of the discharge, the Ne^*^ signal from the FIG 393.3 mm downstream the crossing point was used. Moreover, the UV laser was detected by a photodiode at the entrance of the main chamber. Data points acquired with poor discharge conditions and laser power lower than 80% of the average over the whole scan were discarded. Since the densities of the two beams were not quantitatively determined, we could not extract absolute cross-sections. Relative experimental cross-sections for C(^3^P_0_) + *o*-*, n*-D_2_ → C(^3^P_j = 1, 2_) + *o*-*, n*-D_2_ inelastic collisions were obtained by accumulating at each angle signals from several thousand beam pulses. They were normalized by the sum of the experimental cross-sections in the energy ranges 22 −114 cm^−1^ for C(^3^P_0_) → C(^3^P_1_) transition, and 26 – 165 cm^−1^ for C(^3^P_0_) → C(^3^P_2_) transition. All the plotted vertical error bars represent statistical fluctuations at a 95% confidence interval.

### Theory

The full set of electronic PESs is identical for C-H_2_ and C-D_2_ systems and depends only on the mutual distances of the three atoms involved. Hence, in our scattering calculations of C(^3^P_*j*_) with *o*- and *p*-D_2_ we employed the most recent highly-correlated C(^3^P_*j*_)-H_2_ PESs of Kłos et al. (2018) that were computed with the explicitly correlated multireference configuration interaction method (ic-MRCI-F12) (Shiozaki and Werner, 2011, 2013; Shiozaki et al., 2011) with large atomic basis set. Briefly, the interaction of the open-shell C(^3^P_*j*_) atom with the H_2_ molecule gives rise to two adiabatic potentials of the A″ symmetry (wave function anti-symmetric with respect to the reflection in three-atomic plane) and one of A′ symmetry (symmetric with respect to the in-plane reflection). The two adiabatic potentials were diabatized before the dynamical calculations and the diabatization procedure provided additional off-diagonal coupling. We refer the readers to Kłos et al. (2018) for more details about *ab initio* calculations of the PESs. The geometry of the H_2_ molecule in the C-H_2_ PESs is fixed at the *r*_0_ = 0.767 Å distance corresponding to an average over the lowest vibrational state *v* = 0 and considered as a rigid rotor. As we substitute both H atoms with D isotopologs, we do not need to modify center of mass of the diatomic as the molecule is still homonuclear. In a case of a single isotope substitution to form an HD molecule, one would need to shift the center of mass accordingly. The rigid rotor approach for C-H_2_ and C-D_2_ can use the same set of rigid rotor 2-dimensional PESs assuming that we neglect any mass effects in description of H_2_ and D_2_ that can change slightly the equilibrium distance. The difference between vibrationally averaged distance *r*_0_ for the D_2_ molecule and H_2_ molecule is only about 0.008 Å. Therefore, within these assumptions and approximations we can use our C-H_2_ PESs for the C-D_2_ system.

**Figure 6:**
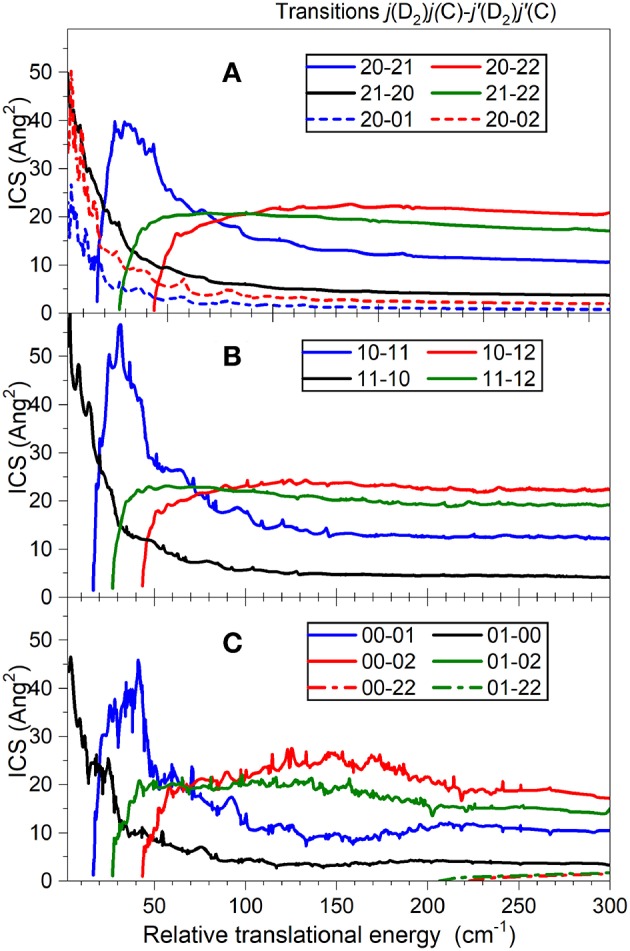
Theoretical cross-sections for C(^3^P_*j*_*C*__) + D_2_(*j*_D_2__) → C(^3^${\text{P}}_{{{\text{j}}^{\prime }}_{C}}$) + D_2_(*j'*_D_2__) inelastic collisions. Transitions are labeled {*j*_D_2__ *j*_C_ – *j'*_D_2__ *j'*_C_}. **(A)** *ortho*-D_2_ (*j*_*D*_2__ = 2); **(B)** *para*-D_2_ (*j*_*D*_2__ = 1); **(C)** *ortho*-D_2_ (*j*_*D*_2__ = 0).

To obtain integral cross-sections we solved Close-Coupling equations of Arthurs and Dalgarno (1960) using the HIBRIDON package^1^. For the C(^3^P_*j*_)-D_2_ scattering we use the reduced mass of 3.0158368 a.m.u. The rotational constant of the D_2_ molecule (Huber and Herzberg, 1979) is *B*_0_ = 29.9037 cm^−1^ and the spin-orbit energy levels of C(^3^P_*j*_) atom (Cooksy et al., 1986; Yamamoto and Saito, 1991), listed in NIST basic atomic spectroscopic data are 0, 16.42 and 43.41cm^−1^ for C(^3^P_0_), C(^3^P_1_), and C(^3^P_2_), respectively. The Close-Coupling equations are propagated using hybrid Alexander-Manolopoulos propagator from the initial distance of *R* = 1.0 to 80 a_0_. The cross-sections were checked for convergence with respect to the inclusion of a sufficient number of partial waves and energetically closed channels. The *o*-D_2_ basis included all levels with a rotational quantum number *j*_*D*_2__ ≤ 6. State-to-state excitation cross-sections were obtained between all the fine-structure levels of C(^3^P) over the collision energy range relevant to the experiments (0 – 800 cm^−1^) on the grid of energies with a step of 0.1 cm^−1^ (see Figure 6). C-H_2_ and C-D_2_ calculations differ by the different energy structure of the partners and by a different reduced mass.

## Results and Discussion

**Figure 7:**
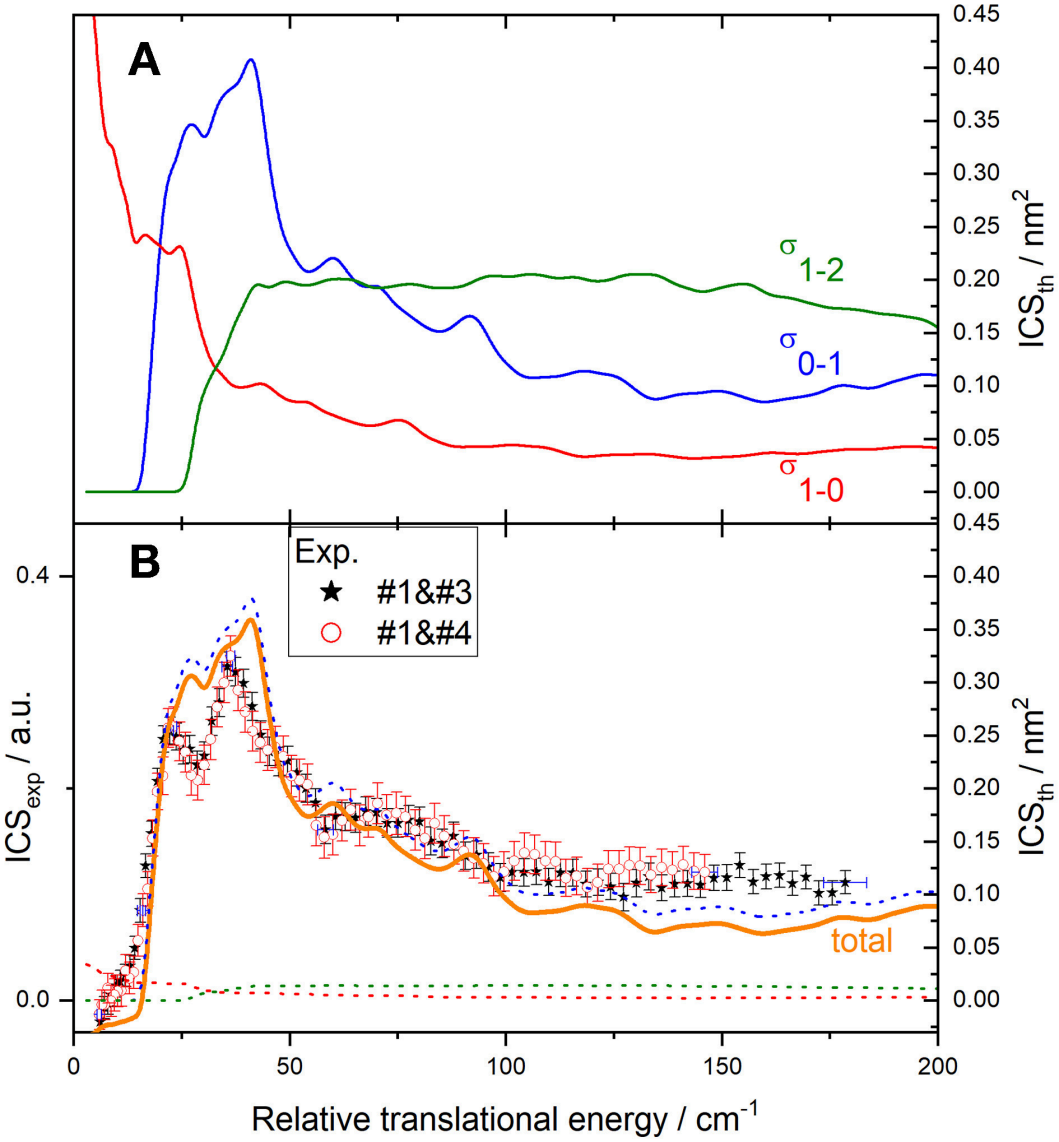
Experimental and theoretical cross-sections for C(^3^P_0_) + *o*-D_2_ → C(^3^P_1_) + *o*-D_2_ inelastic collisions. **(A)** theory convoluted to experimental resolution for individual transitions of C(^3^P_j_) with *o*-D_2_(*j*_*D*_2__ = 0): C(^3^P_0_) → C(^3^P_1_) (blue solid line), C(^3^P_1_) → C(^3^P_2_) (green solid line), and C(^3^P_1_) → C(^3^P_0_) (red solid line); **(B)** experiment (red open circles in the conditions #1 and #4 and black stars in the conditions #1 and #3 described in Table 1); theoretical contributions of transitions C(^3^P_0_) → C(^3^P_1_) (blue dotted line), C(^3^P_1_) → C(^3^P_2_) (green dotted line), C(^3^P_1_) → C(^3^P_0_) (red dotted line), and total (orange solid line), assuming a 7% initial population of C(^3^P_1_): C(^3^P_2_) is populated by C(^3^P_0_) → C(^3^P_1_) (main contribution) and depopulated by C(^3^P_1_) → C(^3^P_2_) and C(^3^P_1_) → C(^3^P_0_) transitions, resulting in a total ICS_tot_ = 0.93 σ_0−1_ – 0.07 (σ_1−2_ + σ_1−0_). To allow for easier comparison, experimental ICS scale is adjusted such that the area in the [22 – 116] cm^−1^ range is the same as for the theoretical ICS calculated. Vertical error bars represent the statistical uncertainties at the 95% confidence interval; each point corresponds to 2020 (experimental conditions #1 and #4) and 1680 (experimental conditions #1 and #3) laser shots per angle, scanning the beam intersection angle between 90° and 13°, with −1° decrement. The plotted error bars on energy are estimated from velocity and crossing angle uncertainties.

**Figure 8:**
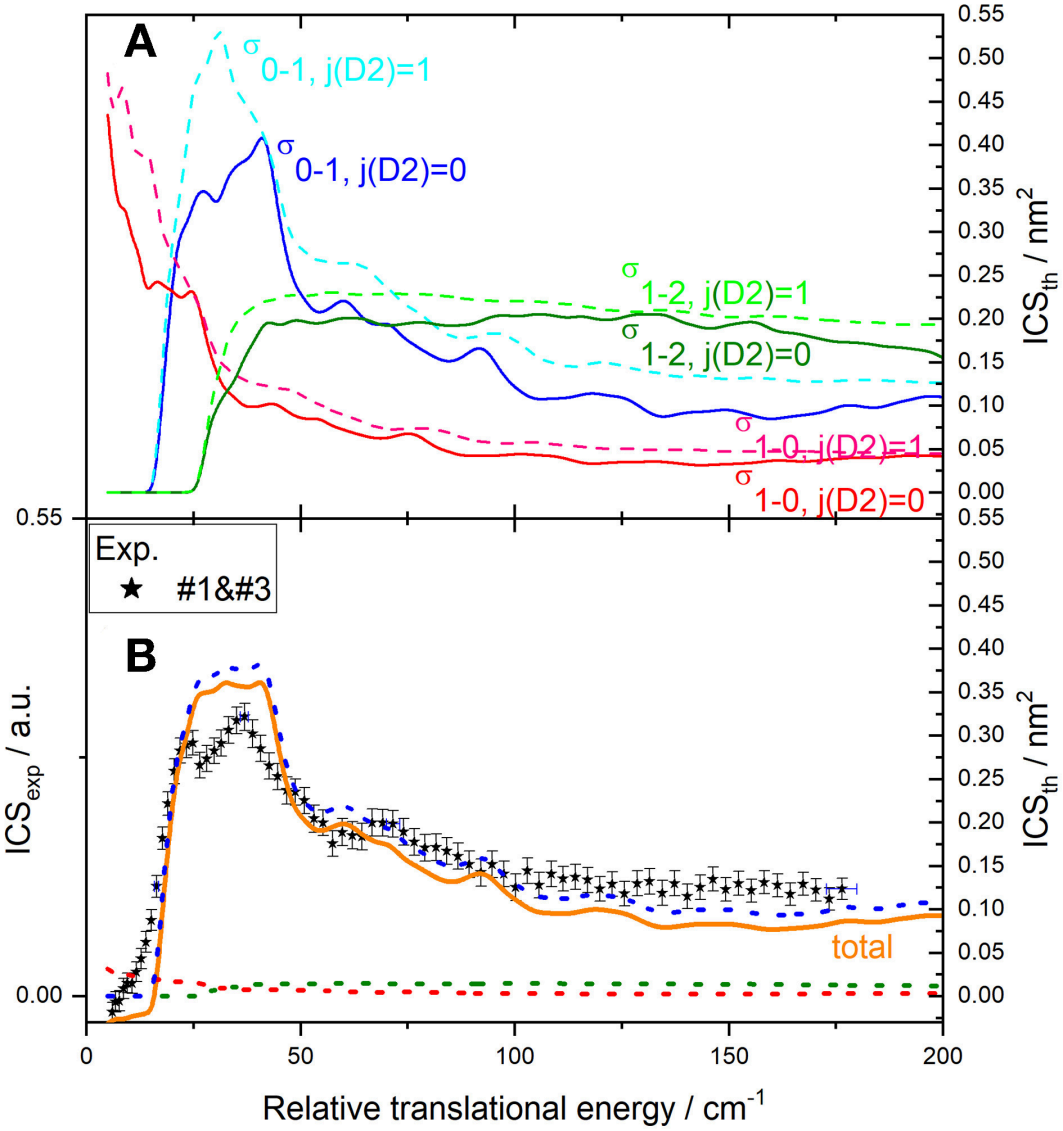
Experimental and theoretical cross-sections for C(^3^P_0_) + *n*-D_2_ → C(^3^P_1_) + *n*-D_2_ inelastic collisions. **(A)** theory convoluted to experimental resolution for individual transitions of C(^3^P_j_) with D_2_(*j*_*D*_2__ = 0) and D_2_(*j*_*D*_2__ = 1): C(^3^P_0_) → C(^3^P_1_) (blue solid line and cyan dashed line), C(^3^P_1_) → C(^3^P_2_) (green solid line and dashed line), C(^3^P_1_) → C(^3^P_0_) (red solid line and pink dashed line); **(B)** experiment (black stars in the conditions #1 and #3 described in Table 1); theoretical contributions of transitions C(^3^P_0_) → C(^3^P_1_) (blue dotted line), C(^3^P_1_) → C(^3^P_2_) (green dotted line), C(^3^P_1_) → C(^3^P_0_) (red dotted line), and total (orange solid line), assuming that *n*-D_2_ is composed of 33% of *j*_D_2__ = 1 and 66% of *j*_D_2__ = 0 and a 7% initial population of C(^3^P_1_): C(^3^P_1_) is populated by C(^3^P_0_) → C(^3^P_1_) (main contribution) and depopulated by the C(^3^P_1_) → C(^3^P_2_) and C(^3^P_1_) → C(^3^P_0_) transitions by collision with D_2_(*j*_*D*_2__ = 0) and D_2_(*j*_*D*_2__ = 1), resulting in a total ICS_tot_ = 0.93 (0.33σ_0−1, j(_D__2_) = 1_ + 0.66 σ_0−1, j(_D__2_) = 0_) – 0.07 {0.33(σ_1−2, j(_D__2_) = 1_ + σ_1−0, j(_D__2_) = 1_) + 0.66(σ_1−2, j(_D__2_) = 0_ + σ_1−0, j(_D__2_) = 0_)}. To allow for easier comparison, experimental ICS scale is adjusted such that the area in the [22 – 114] cm^−1^ range is the same as for the theoretical ICS calculated. Vertical error bars represent the statistical uncertainties at the 95% confidence interval; each point corresponds to 2060 laser shots per angle, scanning the beam intersection angle between 90° and 14°, with −1° decrement. The plotted error bars on energy are estimated from velocity and crossing angle uncertainties.

**Figure 9:**
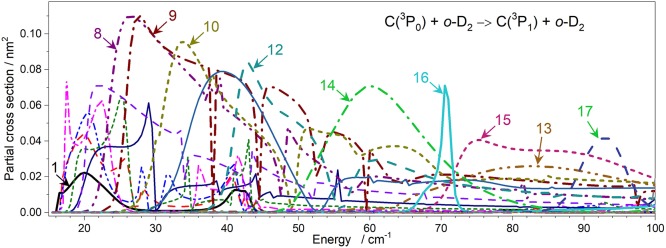
Partial waves calculated for the transition C(^3^P_0_) + *o*-D_2_ → C(^3^P_1_) + *o*-D_2_. Some of the most prominent partial waves are labeled with their total angular momentum value, *J*_t_.

**Figure 10:**
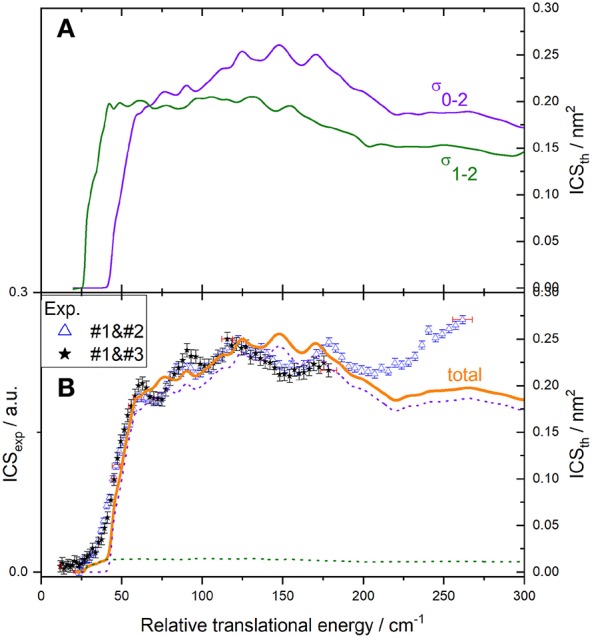
Experimental and theoretical cross-sections for C(^3^P_0__or1_) + *o*-D_2_ → C(^3^P_2_) + *o*-D_2_ inelastic collisions. **(A)** theory convoluted to experimental resolution for individual transitions: C(^3^P_0_) → C(^3^P_2_) (violet solid line), C(^3^P_1_) → C(^3^P_2_) (green solid line); **(B)** experiment (blue open triangles in the conditions #1 and #2 and black stars in the conditions #1 and #3 described in Table 1); theoretical contributions of transitions C(^3^P_0_) → C(^3^P_2_) (violet dotted line), C(^3^P_1_) → C(^3^P_2_) (green dotted line) and total (orange solid line), assuming a 7% initial population of C(^3^P_1_): C(^3^P_2_) is populated by C(^3^P_0_) → C(^3^P_2_) (main contribution), and C(^3^P_1_) → C(^3^P_2_) excitation transitions, resulting in a total ICStot = 0.93σ_0−2_ + 0.07σ_1−2_. To allow for easier comparison, experimental ICS scale is adjusted such that the area in the [26 – 167] cm^−1^ range is the same as for the theoretical ICS calculated. Vertical error bars represent the statistical uncertainties at the 95% confidence interval; each point corresponds to 630 (experimental conditions #1 and #2) and 1500 (experimental conditions #1 and #3) laser shots per angle, scanning the beam intersection angle between 90° and 13° (experimental conditions #1 and #2) or 20° (experimental conditions #1 and #3), with −1° decrement. The plotted error bars on energy are estimated from velocity and crossing angle uncertainties.

**Figure 11:**
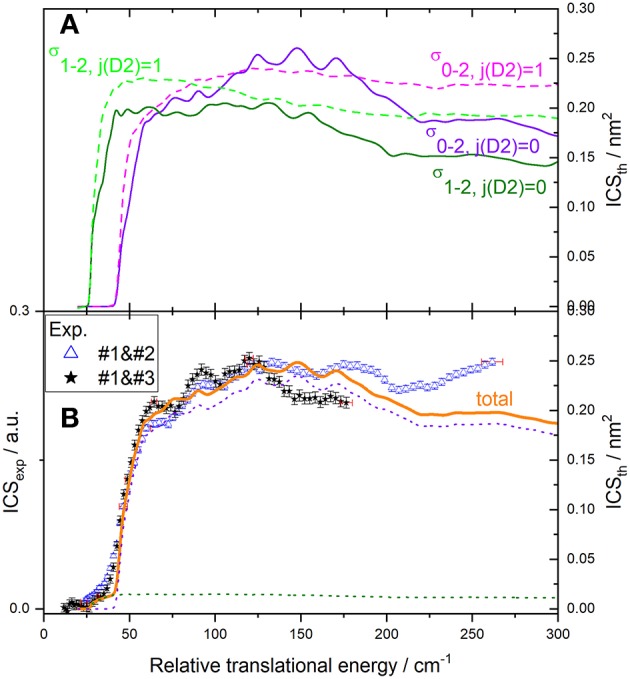
Experimental and theoretical cross-sections for C(^3^P_0 or 1_) + *n*-D_2_ → C(^3^P_2_) + *n*-D_2_ inelastic collisions. **(A)** theory convoluted to experimental resolution for individual transitions of C(^3^P_j_) with D_2_(*j*_*D*_2__ = 0) and D_2_(*j*_*D*_2__ = 1): C(^3^P_0_) → C(^3^P_2_) (violet solid line and magenta dashed line), C(^3^P_1_) → C(^3^P_2_) (green solid line and dashed line); **(B)** experiment (blue open triangles in the conditions #1 and #2 and black stars in the conditions #1 and #3 described in Table 1); theoretical contributions of transitions C(^3^P_0_) → C(^3^P_2_) (violet dotted line), C(^3^P_1_) → C(^3^P_2_) (green dotted line), and total (orange solid line), assuming that *n*-D_2_ is composed of 33% of *j*_D_2__ = 1 and 66% of *j*_D_2__ = 0 and a 7% initial population of C(^3^P_1_): C(^3^P_2_) is populated by C(^3^P_0_) → C(^3^P_2_) (main contribution) and C(^3^P_1_) → C(^3^P_2_) excitation transitions by collision with D_2_(*j*_*D*_2__ = 0) and D_2_(*j*_*D*_2__ = 1), resulting in a total ICS_tot_ = 0.93 (0.33σ_0−2, j(_D__2_) = 1_ + 0.66 σ_0−2, j(_D__2_) = 0_) + 0.07 (0.33σ_1−2, j(_D__2_) = 1_ + 0.66σ_1−2, j(_D__2_) = 0_). To allow for easier comparison, experimental ICS scale is adjusted such that the area in the [26 – 165] cm^−1^ range is the same as for the theoretical ICS calculated. Vertical error bars represent the statistical uncertainties at the 95% confidence interval; each point corresponds to 980 (experimental conditions #1 and #2) and 1150 (experimental conditions #1 and #3) laser shots per angle, scanning the beam intersection angle between 90° and 13° (experimental conditions #1 and #2) or 20° (experimental conditions #1 and #3), with −1° decrement. The plotted error bars on energy are estimated from velocity and crossing angle uncertainties.

**Figure 12:**
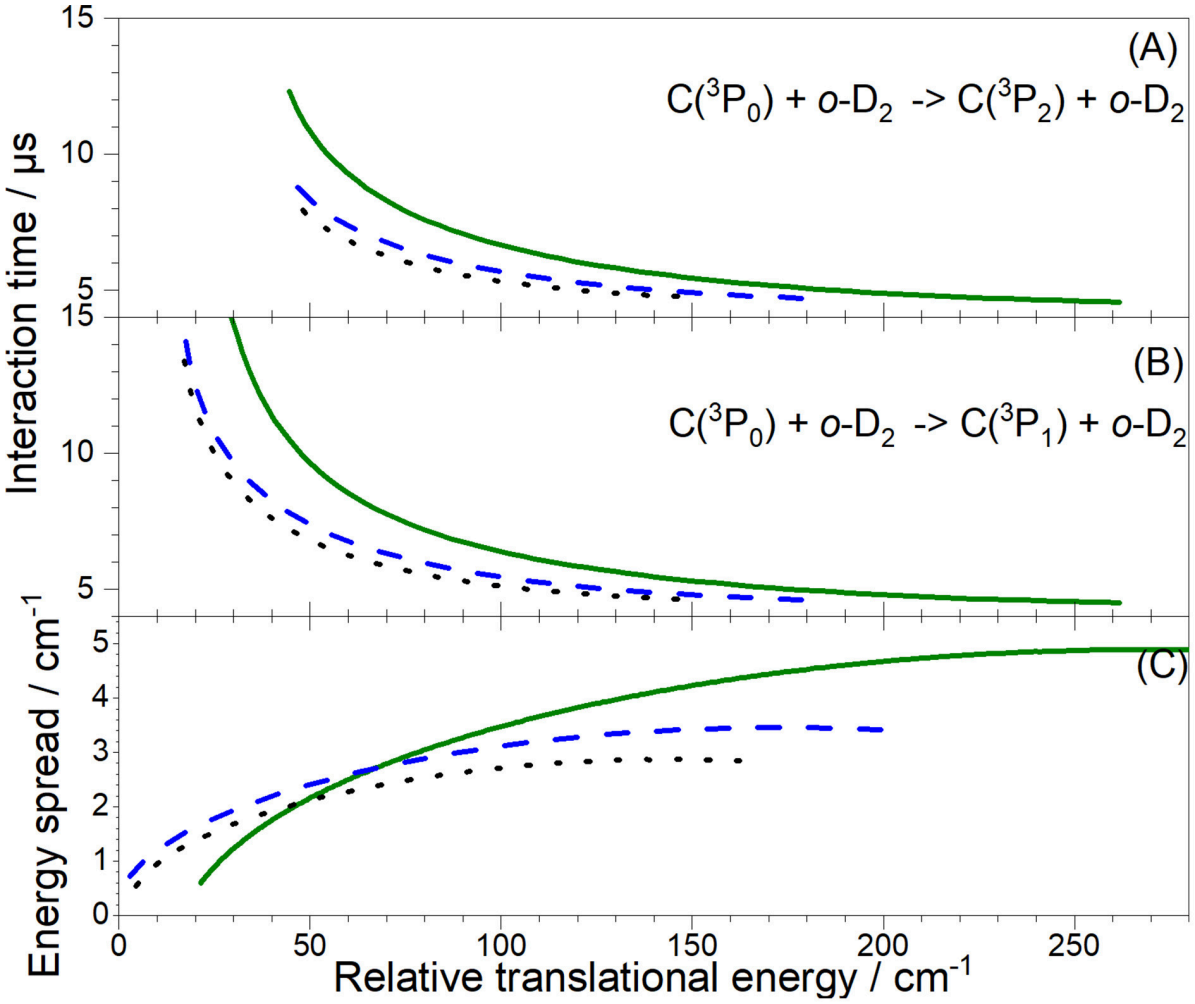
Interaction time **(A,B)**, and collision energy spread **(C)**, calculated for the experimental conditions of previous Figures of the C + *o*-D_2_ collision energy transfers: in blue dashed lines for the experimental conditions #1(C) and #3(D_2_, 45 K), in olive solid lines for #1(C) and #2(D_2_, 100 K) and in black dot lines for #1(C) and #4(D_2_, 10 K). Note that the angular spread is the main source of collision energy spread. All data are plotted as a function of the collision energy, *E*_*T*_. For the interaction time, a forward distribution is considered: **(A)** for the C(^3^P_0_) → C(^3^P_1_) transition and **(B)** for the C(^3^P_0_) → C(^3^P_2_) transition, same labels as for the collision energy spread.

As expected, the excitation functions, i.e., the variation of the ICSs as a function of relative translation energy, for C(^3^P_0_) + D_2_ → C(^3^P_1_) + D_2_ and C(^3^P_0_) + D_2_ → C(^3^P_2_) + D_2_ (Figures 7–11) all exhibit a threshold behavior corresponding to the spin-orbit energy differences: *E*(^3^P_1_) – *E*(^3^P_0_) = 16.42 cm^−1^ and *E*(^3^P_2_) – *E*(^3^P_0_) = 43.41 cm^−1^. In Figure 6 are displayed the calculated ICSs for all transitions which may contribute to the observed signals. It is worth noting that the ICSs for relaxation transitions rapidly decrease at high energies. To allow for comparison with experimental data, these ICSs were convoluted with the experimental energy resolution (Figure 12).

### C(^3^P_0_) + *o*(*n*)-D_2_ → C(^3^P_1_) + *o*(*n*)-D_2_ Transitions

Results for the C(^3^P_0_) excitation by collision with *o*- and *n*-D_2_ are displayed in Figures 7, 8, respectively. The convoluted theoretical ICSs for involved transitions, given in the upper panels, are also displayed in the lower panels weighted by their estimated contributions to the total process: due to the presence of a small amount of C(^3^P_1_), transitions C(^3^P_1_) → C(^3^P_0_) and C(^3^P_1_) → C(^3^P_2_) need to be taken into account. The resulting total ICS can then be compared to experimental results.

For collisions with *o*-D_2_ (Figure 7), two sets of experiments were achieved, under different D_2_-beam conditions, with different velocities: consequently, data for a given energy have been acquired at different crossing angles. The excellent agreement between both experimental sets ensures the absence of any bias in the method used to recover the ICS from the REMPI signal, which involves estimating the mean interaction time 〈Δ*t*〉, which depends on the crossing angle (**Figure 12**). Only transitions involving D_2_ (*j*_*D*_2__ = 0) are taken into account in the overall convoluted theoretical ICS, which indeed is not the case for collisions with *n*-D_2_ (Figure 8), where transitions involving D_2_ (*j*_*D*_2__ = 1) also need to be taken into account with relative weights of 2/3 and 1/3, respectively.

Resonance structures observed experimentally are very similar to the theoretical ones, however slightly shifted in energy. The agreement is better for *o*-D_2_ than for *n*-D_2_, since more contributions are involved in the latter case. In particular, the first two peaks at *ca*. 26.5 and 41.3 cm^−1^ for *j*_D_2__ = 0 are well resolved in both theory and experiment for *o*-D_2_ (Figure 7A), but are blurred out by the peak at *ca*. 31 cm^−1^ for *j*_D_2__ = 1 for *n*-D_2_ (Figure 8B). In Figure 9 are displayed partial cross sections for C(^3^P_0_) → C(^3^P_1_) corresponding to partial waves with total angular momenta *J*_t_ from 1 to 20 that contribute within the 16–100 cm^−1^ total energy range. The peaks around 20 and 40 already appear at *J*_t_ = 1 (thick black solid line) and are associated with opening of carbon spin orbit channels. The first lowest partial waves form sharp peaks around 20 cm^−1^, whereas a broader peak around 30–40 cm^−1^ is due to the build-up of partial waves with *J*_t_ = 8–12. The peak around 60 cm^−1^ comes from a shape resonance for *J*_t_ = 14. A sharp shape resonance also appears for *J*_t_ = 17 near 90 cm^−1^.

### C(^3^P_0_) + *o*(*n*)-D_2_ → C(^3^P_2_) + *o*(*n*)-D_2_ Transitions

Results for the C(^3^P_0_) excitation by collision with *o*- and *n*-D_2_ are displayed in Figures 10, 11. The convoluted theoretical ICSs for involved transitions, given in the upper panels, are also displayed in the lower panels weighted by their estimated contributions to the total process: again, due to the presence of a small amount of C(^3^P_1_), transition C(^3^P_1_) → C(^3^P_2_) need to be taken into account. Note that the collisional de-excitation of C(^3^P_2_) is not taken into account here since its initial population is negligible: furthermore, it would result in a negative offset below threshold not observed. The resulting total ICS can then be compared to experimental results.

In both cases, two sets of experiments were achieved, under different D_2_-beam conditions, with different velocities. Whereas both experimental sets are in good agreement for *o*-D_2_ (Figure 10B) up to 160 cm^−1^, it is not the case for *n*-D_2_ experiments. However, the beam velocity mismatch is larger for C(^3^P_0_) → C(^3^P_2_) experiments (*v*_c_ = 815; *v*_D2_ = 845 and 1180 ms^−1^, resulting in *E*_T_ = 179 and 262 cm^−1^ at χ = 90°) than for C(^3^P_0_) → C(^3^P_1_) experiments (*v*_c_ = 815; *v*_D2_ = 694 and 855 ms^−1^, resulting in *E*_T_ = 146 and 179 cm^−1^ at χ = 90°): at 90°, the interaction time is much shorter than at lower angles: a variation (even small) in its estimated value can result in a larger variation of the ICS value (= *I*_REMPI_/(*v*_*r*_ 〈Δ*t*〉)). It is however worth noting that the increase of the cross-section at high energies is too high to be due to a 〈Δ*t*〉 possible underestimate. It could be due to an energy transfer involving relaxation of D_2_ (*j*_*D*_2__ = 2 → 0): however, ICSs involving such a relaxation are monotonously decreasing (Figure 6), which would imply that their contribution should be much more intense at low energies resulting in a significant positive offset not observed. Furthermore, ICSs corresponding to rotational (de-)excitation of D_2_ are weak (Figure 6). This is due to a weak anisotropy of the PES with respect to D_2_ rotation that leads to a weak coupling between the D_2_ rotational states. It also explains that the magnitudes of the ICSs are similar for all D_2_ rotational state. In addition, the energy splitting between *j*_*D*_2__ = 0 and *j*_*D*_2__ = 2 state of D_2_ is so large that the excitation probability is very small.

Another possibility to be considered is the coupling with the C(^1^D_2_) state: its crossing with triplet PESs is estimated around 300 cm^−1^ by theory. Excitation to the ^1^D_2_ state is closed but the increase of the ICS may be due to indirect coupling that will favor the ^3^P_2_ state over the ^3^P_1_. This would result in a decrease of the ICS for the ^3^P_1_ in the same energy domain (not probed in the present experiments).

The cross-sections observed are different in shape, but almost similar in average. Resonances are less visible for the C(^3^P_0_) → C(^3^P_2_) than for C(^3^P_0_) → C(^3^P_1_) ICS: actually, there are many more resonances for the 0–2 transition than for 0–1, especially for *j*_D_2__ = 1, resulting in a smoother shape. On average, their amplitudes differ by a factor of ~2. Similar differences were found for the O–H system (Lique et al., 2018). The fact that the C(^3^P_0_) – C(^3^P_2_), C(^3^P_1_) – C(^3^P_2_), and C(^3^P_0_) – C(^3^P_1_) transitions have more of less the same magnitude may be partly explained by the huge well depth that would lead to a significant redistribution of the flux over all open C-atom spin-orbit states.

### Comparison of C–D_2_ With C–H_2_ and C–He Systems

**Figure 13:**
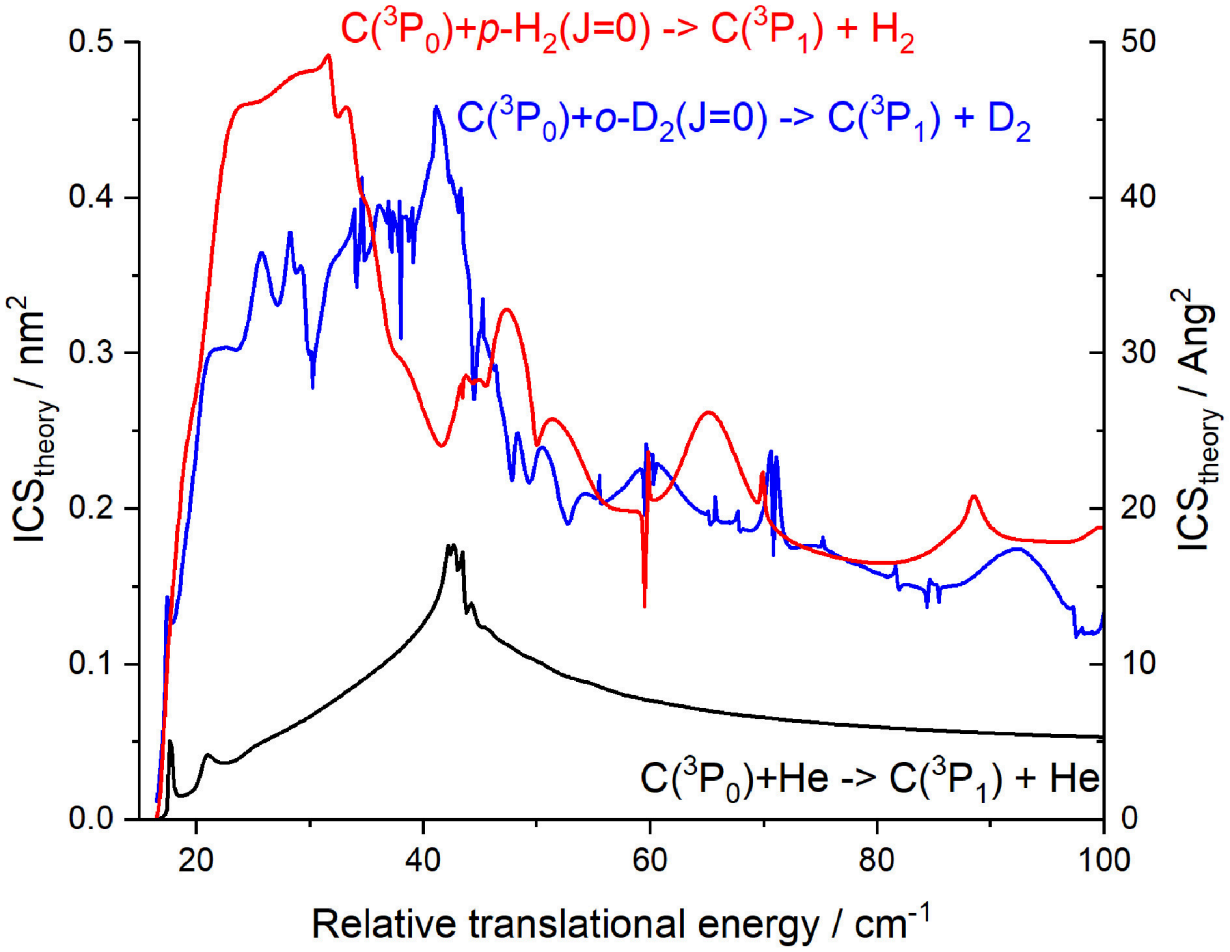
Integral cross sections calculated for C(^3^P_0_) + M → C(^3^P_1_) + M with M = He (black solid line) (Bergeat et al., 2018), *p*-H_2_ (red solid line) (Kłos et al., 2018), and *o*-D_2_(blue solid line).

As shown in Figure 13, there is a great difference between C–He and C–H_2_ (D_2_) systems, due to quite different excitation processes. For C–He, the interaction is a weak van der Waals forces and the excitation is thus essentially governed by spin-orbit coupling (Bergeat et al., 2018). For C–H_2_ (D_2_), the interaction consists in a deep potential well and the system can then be chemically bound and collisional energy transfers therefore are much more efficient, as clearly shown in Figure 13. However, there is a decrease in the C(^3^P_0_) – C(^3^P_1_) ICSs around 50 cm^−1^ for all systems, essentially due to the opening of the C(^3^P_0_) – C(^3^P_2_) excitation. Concerning C–D_2_ and C–H_2_ systems, apart the resonances, ICSs are expected to be similar when no rotational (de-)excitation of H_2_ (D_2_) is involved as can be seen in Figure 13.

## Conclusion

In this work, we have presented a joint theoretical and experimental study of the spin-orbit transitions of C(^3^P_*j*_) by collisions with D_2_. Integral cross-sections have been experimentally observed in a crossed beam apparatus down to collision energies below *ca*. 5.5 cm^−1^ (66 J mol^−1^) and compared to state-of-the-art non-Born-Oppenheimer quantum dynamical calculations for C(^3^P_0_) → C(^3^P_1_) and C(^3^P_0_) → C(^3^P_2_) transitions. Results have been compared to similar systems previously studied (C(^3^P_*j*_) + H_2_, and He) (Bergeat et al., 2018; Kłos et al., 2018). The overall agreement obtained, in particular for C(^3^P_*j*_) + H_2_ and C(^3^P_*j*_) + D_2_ for which calculations are performed using the same potential energy surfaces, confirms the validity of these potentials which can be confidently used to compute (de-)excitation rates of C-atoms in the low energy domain relevant to astrophysical modeling of cold molecular clouds. However, the present results validate the low energy data generated from theses PESs but the calculations at higher energy would require to revise the theoretical approach and probably to add the excited state of C-atom.

## Data Availability

All datasets generated for this study are included in the manuscript and are available on request.

## Author Contributions

AB, SM, and CN carried out the experimental measurements and data analysis. FL and JK performed the theoretical calculations. The manuscript was written through contributions of all authors.

### Conflict of Interest Statement

The authors declare that the research was conducted in the absence of any commercial or financial relationships that could be construed as a potential conflict of interest.

## Footnotes

^1^*Hibridon is a Package of Programs for the Time-Independent Quantum Treatment of Inelastic Collisions and Photodissociation* written by M. H. Alexander, D. E. Manolopoulos, H.-J. Werner, and B. Follmeg, with contributions by P. F. Vohralik, D. Lemoine, G. Corey, R. Gordon, B. Johnson, T. Orlikowski, A. Berning, A. Degli-Esposti, C. Rist, P. J. Dagdigian, B. Pouilly, G. van der Sanden, M. Yang, F. de Weerd, S. Gregurick, J. Kłos and F. Lique. Available online at: {http://www2.chem.umd.edu/groups/alexander/hibridon/hib43/}
